# Severe, rapidly progressive, atypical scoliosis with macrocephaly and a de novo phosphatase and tensin homolog missense variant: A case report

**DOI:** 10.1097/MD.0000000000050052

**Published:** 2026-08-07

**Authors:** Chen Liu, Honggen Du, Yinling Sun, Xiaomin Chen, Kaiqi Wang, Junxiang Dong, Shao Chen

**Affiliations:** a The First Affiliated Hospital of Zhejiang Chinese Medical University (Zhejiang Provincial Hospital of Chinese Medicine), Hangzhou, Zhejiang Province, China.

**Keywords:** case report, macrocephaly, *PTEN*, scoliosis, syndromic scoliosis

## Abstract

**Rationale:**

Early-onset, rapidly progressive, markedly asymmetric, or otherwise atypical scoliosis should prompt evaluation for an underlying genetic etiology. Variants in phosphatase and tensin homolog (*PTEN*) are associated with macrocephaly and a broad spectrum of developmental phenotypes; however, their clinical relevance to severe atypical scoliosis remains infrequently described. This case highlights the potential diagnostic value of genetic evaluation in patients with atypical scoliosis accompanied by abnormal head growth.

**Patient concerns:**

A 13-year-3-month-old boy presented with scoliosis that was first recognized at 9 years of age and subsequently progressed markedly. He exhibited early-onset, rapidly progressive, markedly asymmetric scoliosis accompanied by macrocephaly.

**Diagnoses:**

Exome sequencing identified a heterozygous *PTEN* missense variant, NM_000314.8:c.276C>A (p.D92E), classified as pathogenic. Parental testing showed wild-type alleles in both parents, supporting a de novo origin. The overall findings were consistent with severe, rapidly progressive atypical scoliosis accompanied by macrocephaly and a de novo *PTEN* variant.

**Interventions:**

Because the family declined surgical treatment, multimodal conservative management was initiated, consisting of a Chêneau brace worn for 21 hours per day, physiotherapeutic scoliosis-specific exercises for 2 hours per day, and adjunctive manual therapy.

**Outcomes:**

During the 12 months of follow-up, no further radiographic progression was observed, and the trunk appearance remained relatively stable. No major treatment-related adverse events were reported.

**Lessons:**

Early genetic evaluation should be considered in patients with scoliosis characterized by early onset, rapid progression, marked asymmetry, and atypical curve patterns, particularly when accompanied by macrocephaly. A de novo *PTEN* variant may provide an important diagnostic clue in such cases. For patients with severe scoliosis harboring a *PTEN* variant who decline surgery, multimodal conservative treatment may serve as a bridging strategy to achieve short-term curve stability, while continued multidisciplinary assessment, genetic counseling, and long-term surveillance for potential *PTEN*-related manifestations remain essential.

## 1. Introduction

Scoliosis is a common spinal deformity in children and adolescents, and most cases are classified as idiopathic.^[1,2]^ However, when scoliosis presents with early onset, rapid progression, marked asymmetry, long-segment involvement, or an atypical curve pattern, underlying syndromic, neuromuscular, or genetic etiologies should be considered.^[1,2]^ In such patients, reliance solely on the conventional diagnostic framework for adolescent idiopathic scoliosis (AIS) may delay recognition of an underlying systemic disorder.

The phosphatase and tensin homolog (*PTEN*) gene encodes a key regulator of cell growth, proliferation, and the phosphoinositide 3-kinase/protein kinase B signaling pathway. Germline *PTEN* variants are associated with a heterogeneous phenotypic spectrum that includes macrocephaly, developmental abnormalities, hamartomatous overgrowth, and tumor predisposition.^[3–6]^ Macrocephaly is generally defined as an occipitofrontal circumference greater than 2 standard deviations above the mean for age and sex.^[7]^ Although skeletal abnormalities have been described in *PTEN*-related disorders, severe, rapidly progressive scoliosis with an atypical curve pattern as the major presenting problem is uncommon.^[3–6]^ Here, we report the case of a boy with severe, rapidly progressive atypical scoliosis, macrocephaly, and a de novo *PTEN* missense variant to emphasize the value of early genetic evaluation in atypical scoliosis and to discuss the short-term bridging role of multimodal conservative management when surgery is declined.

## 2. Case presentation

This case report was prepared according to the CARE guidelines. The patient in this study was anonymized. Written informed consent for the publication of this case report and accompanying clinical images was obtained from the patient’s legal guardian. Written consent was obtained from the patient after an age-appropriate explanation of the purpose of publication and anonymization of personal information. This study was approved by the Medical Ethics Committee of the First Affiliated Hospital of Zhejiang Chinese Medical University (Zhejiang Provincial Hospital of Chinese Medicine) (approval no. 2024-KLS-504-03).

**Figure 1.**
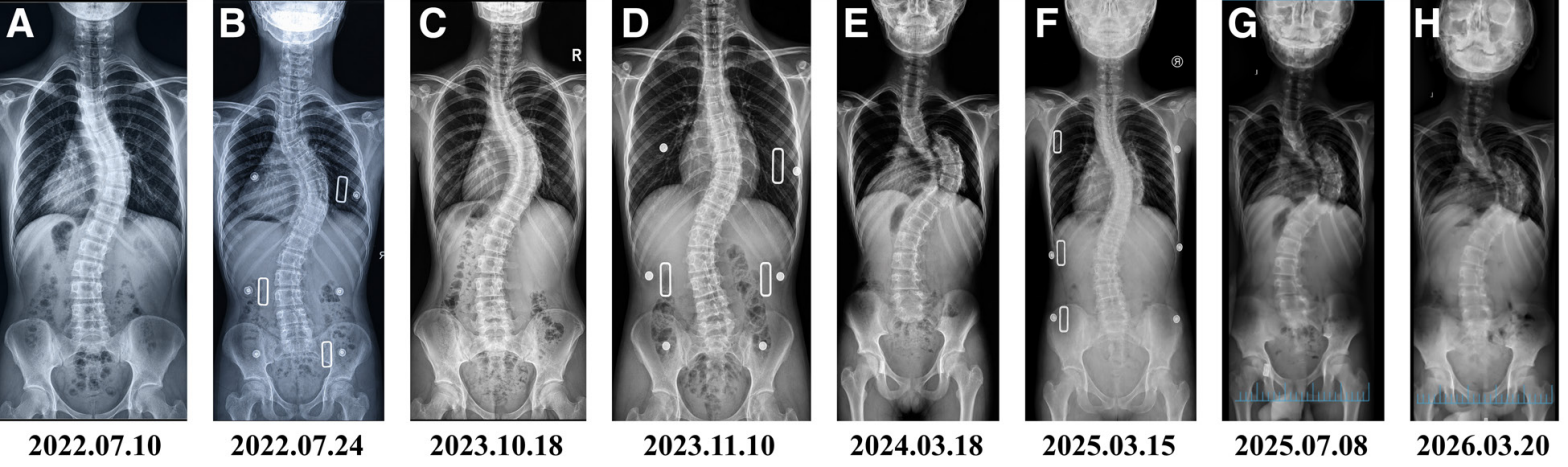
Standing, posteroanterior, whole-spine radiographs showing the progression and short-term follow-up of the spinal deformity. At initial presentation, the thoracic and lumbar Cobb angles were 48° and 26°, respectively. Before the start of the current treatment, the thoracic Cobb angle had progressed to 74° and the lumbar Cobb angle to 35°. The start date of the treatment was March 15, 2025. After 12 months of multimodal, conservative management consisting of a Chêneau brace, physiotherapeutic scoliosis-specific exercises, and adjunctive manual therapy, the thoracic and lumbar Cobb angles remained at 74° and 35°, respectively, with no further radiographic progression. Panels (A–H) indicate the time course from October 7, 2022, to March 3, 2026.

A 13-year-3-month-old boy presented with scoliosis that had been recognized for over 4 years and showed obvious progression. The scoliosis was first noted at the age of 9 years, when standing whole-spine radiographs demonstrated a thoracic Cobb angle of 48° and a lumbar Cobb angle of 26°. He was treated with bracing; however, the deformity continued to progress. Prior to treatment, the thoracic and lumbar Cobb angles increased to 74° and 35°, respectively (Fig. 1A–E; Table 1).

**Table 1:** Clinical timeline (initial presentation, investigations, treatments, follow-up).

Date	Clinical procedure/event
July 10, 2022	Initial radiographic assessment; scoliosis recognized
July 24, 2022	X-rays, brace
October 18, 2023	X-rays
November 10, 2023	X-rays, brace
March 18, 2024	X-rays
March 15, 2025	X-rays, brace (21 h/d), PSSE (2 h/d), and adjunctive manual therapy (once a week)
July 8, 2025	X-rays
July 31, 2025	Gene detection
September 8, 2025	Identification of a de novo *PTEN* missense variant
March 20, 2026	X-rays

This clinical timeline is divided into 10 time periods, including the specific times of initial presentation, imaging studies, treatment, diagnosis confirmation, and follow-up.

PSSE = physiotherapeutic scoliosis-specific exercises, *PTEN* = phosphatase and tensin homolog.

The patient had no history of spinal trauma or surgery. Further review of the perinatal and developmental histories indicated that third-trimester prenatal ultrasonography showed lateral ventricular widening and a relatively large head circumference. No prenatal genetic diagnoses were made. After birth, the patient had a large head; however, exact neonatal measurements were not available. There was a history of delayed posterior fontanel closure. The family reported mild concerns regarding learning and cognition; however, the Combined Raven’s test showed an intelligence quotient of 98 (P44), which did not indicate definite intellectual impairment.

**Figure 2.**
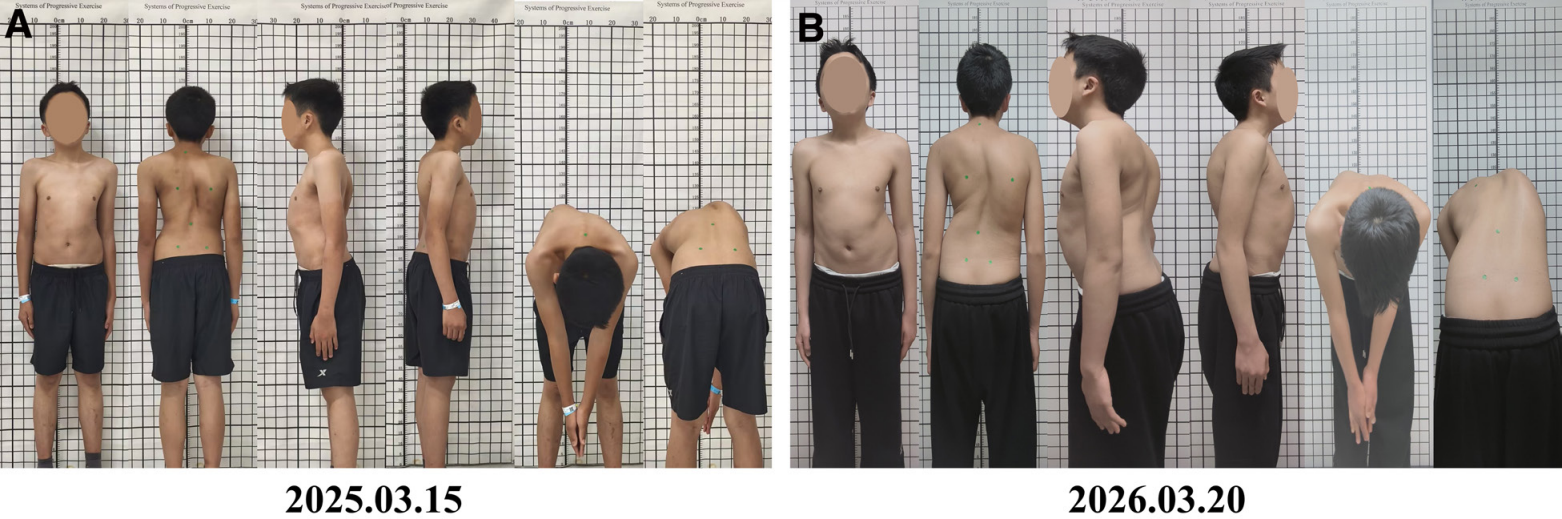
A 13-year-3-month-old boy with scoliosis first noted at 9 years of age, followed by obvious progression. Comparison of body posture (A) before treatment and (B) at the 12-month follow-up period. Posture was improved compared with baseline. The initial angle of trunk rotation (ATR) values were 21° at the thoracic level and 7° at the lumbar level, decreasing to 17° (thoracic) and 5° (lumbar) after treatment at follow-up.

Physical examination indicated shoulder imbalance, trunk shift, and marked thoracolumbar asymmetry, with a positive Adam forward bending test result. The patient had a double-curved deformity with a major thoracic curve. The angle of trunk rotation was 21° at the thoracic and 7° at the lumbar levels, and the occipitofrontal circumference was 60 cm (Fig. 2A).

**Figure 3.**
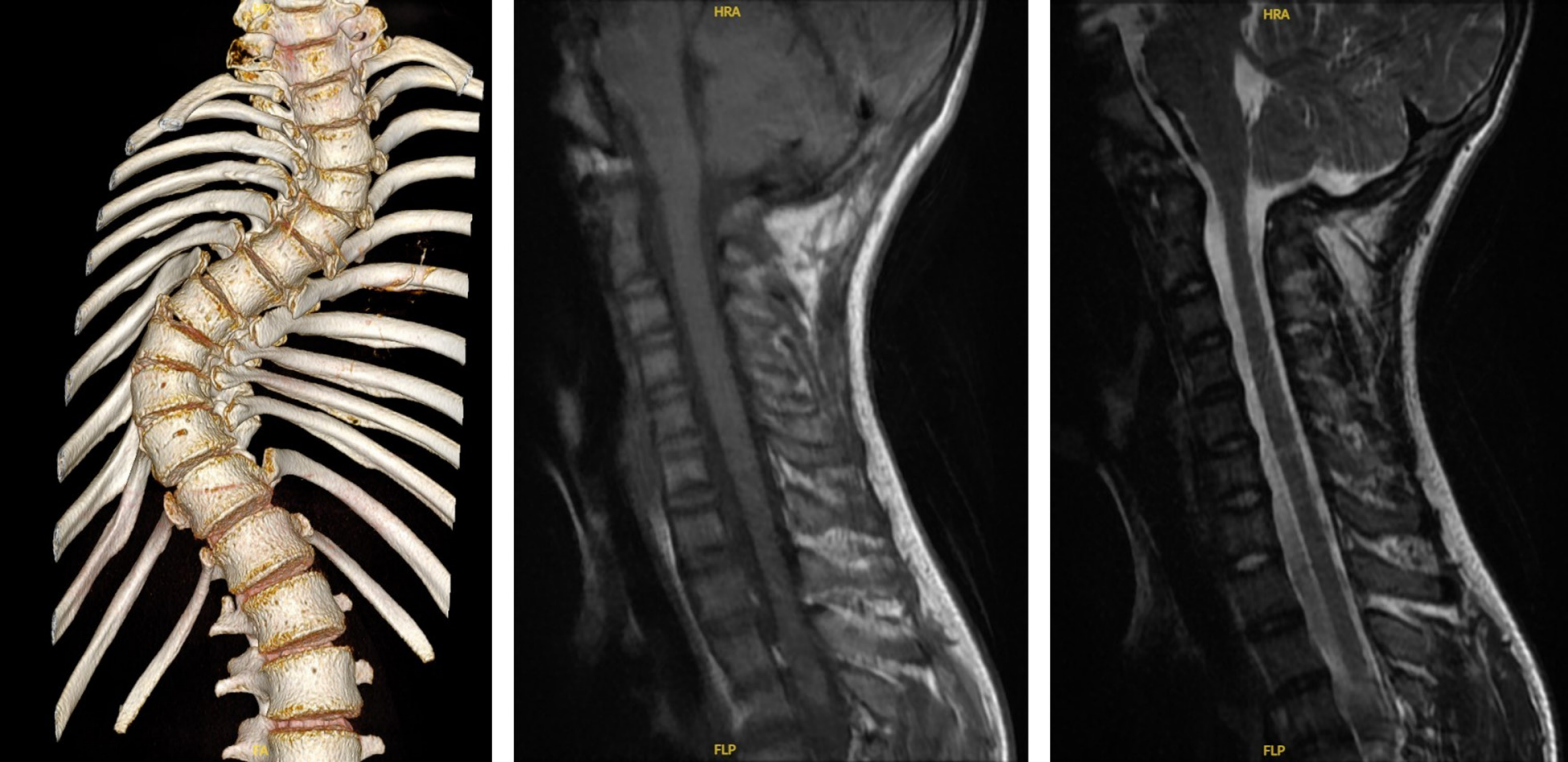
Three-dimensional, reconstructed computed tomography scan of the thoracic spine and cervical magnetic resonance imaging showing no obvious congenital vertebral malformations or cervical intraspinal abnormalities. FLP = foot–left–posterior, HRA = head–right–anterior.

Standing whole-spine radiography confirmed severe scoliosis. At initial presentation, the thoracic and lumbar Cobb angles were 48° and 26°, respectively. Before treatment, the thoracic and lumbar Cobb angles had progressed to 74° and 35°, respectively. The thoracic curve extended from T6 to T12, and the lumbar curve extended from T12 to L4. The Risser grade was 2, which indicated ongoing skeletal growth. Three-dimensional, reconstructed computed tomography of the thoracic spine and cervical magnetic resonance imaging showed no obvious congenital vertebral malformations, such as hemivertebrae, and no evidence of Chiari malformation, syringomyelia, tethered cord, or other intraspinal abnormalities (Fig. 3). These features, together with the early onset, rapid progression, marked asymmetry, and atypical pattern, suggested that the case could not be sufficiently explained by routine AIS alone.

Trio-based exome sequencing was conducted because of the atypical scoliosis phenotype, accompanied by macrocephaly and abnormal head growth clues dating back to the prenatal and neonatal periods. Overall, molecular testing reports did not provide a definitive genetic diagnosis. However, a heterozygous, de novo *PTEN* missense variant, NM_000314.8:c.276C>A (p.D92E), located on chr10:89692792, was identified in the proband and was absent in both parents. The reference and mutant read counts in the proband were 139 and 140, respectively, which were consistent with heterozygosity. Parental testing showed only reference alleles in both parents, with reference/mutant read counts of 322/0 in the mother and 269/0 in the father, supporting a de novo event. According to the laboratory’s American College of Medical Genetics and Genomics-based interpretation, this variant was classified as a suspected pathogenic variant, with supporting evidence, including PS2_P, PM2_P, PM5, and PP3_M. This variant was considered phenotypically relevant.

The patient’s family history was unremarkable. Both parents and the patient’s 2 younger sisters were healthy, with no known family history of scoliosis, abnormal head size, developmental abnormalities, confirmed hereditary disease, or tumor predisposition disorders.

Before treatment, the thoracic Cobb angle had reached 74°, indicating severe scoliosis and theoretically warranting surgical evaluation. However, the patient’s family declined surgical treatment. Accordingly, a multimodal conservative regimen consisting of a Chêneau brace prescribed for 21 hours per day, physiotherapeutic scoliosis-specific exercises (PSSE) for 2 hours daily during the treatment period, and adjunctive manual therapy^[8]^ aimed at improving trunk soft tissue condition, postural control, and muscular balance was indicated (Table 1; Fig. 1F).

After 12 months of continuous treatment, repeat standing whole-spine radiographs showed that the thoracic and lumbar Cobb angles had remained at 74° and 35°, respectively, with no further radiographic progression (Fig. 1G and H). The angle of trunk rotation decreased to 17° (thoracic) and 5° (lumbar) after treatment (Fig. 2B). No significant adverse events were observed during the treatment.

The patient and his family expressed satisfaction upon learning that the scoliosis had not progressed during the 12-month follow-up period. As they had been concerned about the rapid progression of the curve before treatment, the stable radiographic findings increased their confidence in continuing brace treatment, PSSE, and manual therapy. They considered the outcome encouraging and hoped that the current management strategy would help maintain spinal stability for as long as possible.

## 3. Discussion

The principal clinical significance of this case is that its course deviated substantially from the expected natural history of routine AIS. Scoliosis was first recognized at 9 years of age, and by 13 years of age, the thoracic curve had progressed to 74°, accompanied by marked asymmetry and an atypical double-curve pattern. In addition, 3-dimensional reconstructed computed tomography of the thoracic spine and cervical magnetic resonance imaging showed no obvious congenital vertebral malformations, such as hemivertebrae or cervical intraspinal abnormalities. Collectively, these findings supported moving beyond a purely “idiopathic scoliosis” framework and pursuing etiologic evaluation from a genetic perspective.

The diagnostic signal was strengthened when the scoliosis phenotype was integrated into a broader developmental history. The patient had prenatal clues, including lateral ventricular widening and a relatively large head circumference, followed by a postnatal history of a large head size and delayed posterior fontanel closure. At the current evaluation, the occipitofrontal circumference was 60 cm, supporting a macrocephalic phenotype in the clinical context. The family history was negative, and parental testing demonstrated wild-type alleles in both parents, supporting a de novo event. Together, these findings suggested a sporadic developmental background rather than ordinary familial scoliosis.

*PTEN*-related disorders are phenotypically heterogeneous and often evolve over time.^[3–6]^ Macrocephaly is one of the most recognizable early clues in children carrying *PTEN* variants.^[3–7]^ In the present case, macrocephaly and abnormal head growth clues were present, whereas neurocognitive evaluation did not demonstrate definite intellectual impairment, and no other clear *PTEN*-related features were identified. Therefore, this patient was intentionally described as having severe, rapidly progressive, atypical scoliosis with macrocephaly and a de novo *PTEN* variant, rather than being classified as having a fully established *PTEN* hamartoma tumor syndrome diagnosis. This interpretation is consistent with the current strength of the available clinical evidence.

From a practical standpoint, this case highlights an important diagnostic message for spine clinicians: atypical scoliosis itself may serve as an entry point for genetic evaluation, even when a complete syndromic phenotype is not yet apparent. In patients with early onset, rapid progression, marked asymmetry, atypical curve patterns, macrocephaly, or an abnormal head growth history, germline genetic testing may help clarify the etiologic context and influence long-term counseling and surveillance strategies.^[3–6]^

The treatment-related interpretation of this case requires caution. At presentation, the thoracic curve had already reached a severe range, and standard surgical evaluation was appropriate. As the family declined surgery, the objective of conservative treatment was not the reversal of the severe deformity but short-term stabilization and preservation of a management window. Under a regimen of 21 hours/day Chêneau bracing, 2 hours/day PSSE, and adjunctive manual therapy, the patient showed no further radiographic progression over 12 months. This short-term stability may be clinically meaningful in a surgery-declined scenario; however, it should not be overinterpreted as evidence of long-term disease modification or as a substitute for surgical assessment.

This study has some limitations. The follow-up period was only 12 months, precluding conclusions regarding medium- or long-term outcomes. Although the clinical phenotype strongly suggested the relevance of the *PTEN* variant, this remains a single case, and a direct causal relationship between the *PTEN* p.Asp92Glu variant and this specific scoliosis pattern cannot be established. No additional *PTEN*-related manifestations had been identified at the time of reporting. Nevertheless, the combination of macrocephaly, prenatal and neonatal clues of abnormal head growth, a de novo *PTEN* variant, and severe, atypical, rapidly progressive scoliosis makes this case clinically compelling and diagnostically informative.

## 4. Conclusion

This case suggests that early genetic evaluation should be considered in patients with severe scoliosis showing early onset, rapid progression, marked asymmetry, and atypical curve patterns, especially when accompanied by macrocephaly or abnormal head growth clues from the prenatal or early postnatal periods. A de novo *PTEN* variant may be an important clue in such cases. When the patient’s family declines surgery, combined Chêneau bracing, PSSE, and adjunctive manual therapy may serve as a short-term bridging strategy, although subsequent management should remain grounded in etiological clarification, genetic counseling, and long-term multidisciplinary follow-up.

## Acknowledgments

The authors wish to acknowledge support from the Zhejiang Provincial “Pioneer” and “Leading Goose” R&D Program under Grant No. 2024C03182, the Zhejiang Provincial Natural Science Foundation Major Project under Grant No. D26H270004, and the Zhejiang Provincial Traditional Chinese Medicine Science and Technology Program Project under Grant No. 2024ZL450.

## Author contributions

**Conceptualization:** Chen Liu, Honggen Du, Shao Chen.

**Data curation:** Chen Liu, Yinling Sun, Xiaomin Chen, Kaiqi Wang.

**Funding acquisition:** Chen Liu, Honggen Du, Shao Chen.

**Investigation:** Chen Liu, Honggen Du, Yinling Sun, Xiaomin Chen, Kaiqi Wang, Junxiang Dong, Shao Chen.

**Supervision:** Junxiang Dong.

**Visualization:** Chen Liu.

**Writing – original draft:** Chen Liu.

**Writing – review & editing:** Honggen Du, Shao Chen.
